# Long-Term Exposure to Low-Dose Di-(2-ethylhexyl) Phthalate Impairs Cholesterol Metabolism in Hepatic Stellate Cells and Exacerbates Liver Librosis

**DOI:** 10.3390/ijerph17113802

**Published:** 2020-05-27

**Authors:** Chun-Ya Lee, Fat-Moon Suk, Yuh-Ching Twu, Yi-Jen Liao

**Affiliations:** 1 School of Medical Laboratory Science and Biotechnology, College of Medical Science and Technology, Taipei Medical University, Taipei 110, Taiwan; s9150921@hotmail.com; 2 Division of Gastroenterology, Department of Internal Medicine, Wan Fang Hospital, Taipei Medical University, Taipei 116, Taiwan; fmsuk@tmu.edu.tw; 3 Department of Internal Medicine, School of Medicine, College of Medicine, Taipei Medical University, Taipei 110, Taiwan; 4 Department of Biotechnology and Laboratory Science in Medicine, School of Biomedical Science and Engineering, National Yang-Ming University, Taipei 112, Taiwan; yctwu@ym.edu.tw

**Keywords:** di-(2-ethylhexyl) phthalate long-term low-dose exposure, cholesterol metabolism, hepatic stellate cells, liver fibrosis

## Abstract

Phthalates are often added to plastic products to increase their flexibility. Di-(2-ethylhexyl) phthalate (DEHP) is one of the most common plasticizers. Previously, a major incident involving phthalate-contaminated foodstuffs occurred, where phthalates were deliberately added to foodstuffs as a substitute for emulsifiers, resulting in a threat to public health. DEHP exposure can cause liver damage and further lead to cancer; however, the effects of long-term exposure to low-dose DEHP on hepatic stellate cells (HSCs) and on liver fibrosis are still unclear. In this study, we showed that chronic exposure to low-dose DEHP results in an accumulation of cholesterol in HSCs by disturbing the cholesterol metabolism and enhancing endogenous cholesterol synthesis. In addition, long-term exposure to low-dose DEHP reduces the sensitivity of HSCs to platelet-derived growth factor BB (PDGF-BB)-induced proliferation by blocking the MAPK pathway. Dysfunction of mitochondrial respiration and induction of caspase 3/PARP-dependent apoptosis were observed in HSCs following chronic, low-dose exposure. The carbon tetrachloride (CCl_4_)-induced liver fibrosis mouse model showed that long-term administration of DEHP significantly promoted liver damage, inflammatory infiltration, cholesterol accumulation, and deposition of hepatic collagen. In conclusion, long-term exposure to low-dose DEHP may perturb the cholesterol metabolism in HSCs and accelerate liver damage and fibrosis.

## 1. Introduction

Phthalates are plasticizers and are the main component of soft polyvinyl chloride plastics; they are widely used to increase flexibility, transparency, and durability [1,2,3]. Di-(2-ethylhexyl) phthalate (DEHP), the most abundantly produced phthalate ester, is commonly added in industrial plastics, food packages, cosmetics, children’s toys, and medical devices, such as haemodialysis tubes and blood storage bags [3,4,5]. Due to its hydrophobicity, DEHP easily leaches into the environment and causes public health threats. Under certain conditions, DEHP can directly or indirectly infiltrate the human body by inhalation, ingestion, and dermal absorption [2,6,7]. When DEHP enters a body, lipases rapidly convert DEHP to its primary metabolic form, monoethylhexylphthalate (MEHP), which is preferentially absorbed by tissues and leads to cancer, cardiovascular disease, metabolic disease, and developmental disorders [8,9,10]. The tolerable daily intake level for the general population as assessed by the European Food Safety Authority and the U.S. Environmental Protection Agency (EPA) is 50 µg/kg and 20 µg/kg of body weight, respectively [11]. The daily exposure of DEHP to the general human population from food and the environment is 30 µg/kg; however, haemodialysis patients suffer higher exposure to DEHP (up to 457 µg/kg) from medical tubes [12].

As an endocrine disruptor, DEHP is of great concern because of its toxic effects on the endocrine system, neural development, reproductive system, and multiple organs, including the liver, kidney, lung, and thyroid gland, which can result in cancer [13,14,15]. Regarding the effects of DEHP exposure to the liver, several studies have indicated that cholesterol metabolism disruption [2,3,16], Kupffer cell activation [17,18], hepatocyte apoptosis [13,19], and promotion of hepatic carcinoma [20] occur following exposure to DEHP. However, the mechanisms of long-term exposure to low-dose DEHP exposure in relation to hepatic stellate cell activation and liver fibrosis are still not fully understood. In chronic liver diseases, the development of fibrosis is the first step towards the progression to cirrhosis, portal hypertension, and liver cancer [21,22,23]. Liver cirrhosis is a major cause of mortality causing a need for liver transplantation worldwide, and its therapeutic options are limited. Hepatic stellate cells (HSCs) are the major profibrogenic cells that produce extracellular matrix proteins in a damaged liver [24]. To further investigate the effects and mechanisms of long-term exposure to low-dose DEHP on HSCs and liver fibrosis, we established HSCs that were exposed to a low-dose of DEHP over a prolonged period, and we analyzed CCl_4_-induced liver damage and fibrosis outcomes in chronic DEHP-exposed mice.

## 2. Materials and Methods

### 2.1. Cells

HSC-T6 cells [25] were cultured in Dulbecco’s modified Eagle’s medium (Gibco BRL, Grand Island, NY, USA) with 1% heat-inactivated foetal bovine serum (HyClone, Logan, UT, USA), penicillin (100 U/mL), streptomycin (100 μg/mL), non-essential amino acids (0.1 mM), and L-glutamine (2 mM) in a humidified incubator with 5% CO_2_. To generate long-term, low-dose, DEHP-exposed HSCs, HSC-T6 cells were exposed to 50 and 100 µM DEHP (Alfa Aesar, USA), and the medium containing DEHP was renewed every 2–3 days. After 3.5 months, long-term, low-dose, DEHP-exposed HSCs were obtained. Cell morphology was recorded by using an Olympus CKX41 microscope (Tokyo, Japan). To study the effect of long-term exposure to low-dose DEHP on PDGF-BB-induced cell proliferation, cells were seeded in 6-well plates (2.5 × 10^5^), and then were treated with 10 ng/mL PDGF-BB (R&D Systems, Minneapolis, MN, USA) for different amounts of time. The cells were then subjected to the following proliferation and Western blot analyses.

### 2.2. Cell Proliferation Assay

Cells (2.5 × 10^3^) were seeded in a 96-well plate. After the indicated treatments, the culture medium in each well was replaced with 100 µl of fresh medium containing 10 µl of 5 mg/mL 3-(4,5-dimethylthiazol-2-yl)-2,5-diphenyltetrazolium bromide (MTT) (Sigma-Aldrich, St. Louis, MO, USA). After 4 h of labelling cells with MTT, the medium in each well was removed and replaced with 100 µl of DMSO; cells were then incubated for 10 min at 37 °C. Samples were mixed, and absorbance was read at 540 nm.

### 2.3. Western Blot

Total protein was extracted from cultured cells by using lysis buffer supplemented with protease and phosphatase inhibitors. The protein concentration was measured by protein assay (Bio-Rad Laboratories, CA), and all 30 μg of samples were analyzed. Cellular proteins were separated by SDS-PAGE and then were transferred onto PVDF membranes. The following antibodies used in this study were purchased from Cell Signaling (Beverly, MA, USA): phospho- and total- MEK, ERK, AKT, JNK, p38, caspase 9, caspase 3, and PARP; and Santa Cruz Biotechnology (Santa Cruz, CA, USA): SR-B1, NPC1, Cyp7a1, HMGCR, SREBP2, and FAs. The immunoblotting signals were normalized to total protein or the signal, resulting from blotting with an α-tubulin antibody (Sigma-Aldrich, St Louis, MO, USA). The bands were visualized using an ECL detection reagent (Millipore Corporation, Billerica, MA, USA).

### 2.4. RNA Extraction, Reverse Transcription, and Real-Time Polymerase Chain Reaction (PCR)

TRIzol Reagent (Ambion, Carlsbad, CA, USA) was used to isolate total RNA. Complementary DNA was produced from 2 μg of RNA using high-capacity cDNA reverse transcription kits (Applied Biosystems, Carlsbad, CA, USA). The specific primers are listed in Table 1. Real-time PCRs were performed with a KAPA SYBR^®^ FAST qPCR Master Mix (KAPA Biosystems, Boston, Massachusetts, USA) and were analyzed on a StepOne System (Applied Biosystems, Foster City, CA, USA). The cycle threshold (Ct) values were exported into Excel worksheets for analyses. Comparative Ct analysis was used to determine the gene expression levels normalized to those of GAPDH.

### 2.5. Flow Cytometry

HSCs were washed with PBS, centrifuged at 1500 rpm for 5 min, and the supernatant was then discarded. The cells were resuspended in 1 mL of FACS buffer (2% FBS in PBS) and were centrifuged again at 2000 rpm for 5 min. The cells were then incubated with Annexin V and 7-AAD for 15 min at 4 °C using the PE Annexin V Apoptosis Detection Kit I (BD Biosciences, San Jose, CA, USA). Stained cells were fixed and then analyzed by a CytoFLEX Flow Cytometer (Beckman Coulter, Indianapolis, IN, USA). A dot plot of 7-AAD fluorescence (y-axis) versus Annexin V fluorescence (x-axis) was prepared.

### 2.6. Seahorse Assay

In vivo cell real-time cellular oxygen consumption rates (OCRs) were measured by an XF24 bioenergetic assay (Seahorse Bioscience, Billerica, MA, USA) according to the manufacturer’s instructions. Briefly, HSC-T6 cells were seeded in an XF24-well plate containing complete medium. After 16 h, the XF24 bioenergetic assay was initiated by removing the exhausted medium and replacing it with sodium-bicarbonate-free DMEM containing 2% FBS. The OCR was detected at a steady state, and oligomycin (1 µM), carbonyl cyanide 4-[trifluoromethoxy] phenylhydrazone (FCCP; 2 μM), and rotenone/antimycin A (AA; 0.5 μM) were added sequentially into the wells to obtain the maximal and nonmitochondrial respiration rates.

### 2.7. Established Long-Term, Low-dose, DEHP-exposed Mice for Carbon Tetrachloride (CCl_4_)-Induced Liver Fibrosis

**Figure 1:**
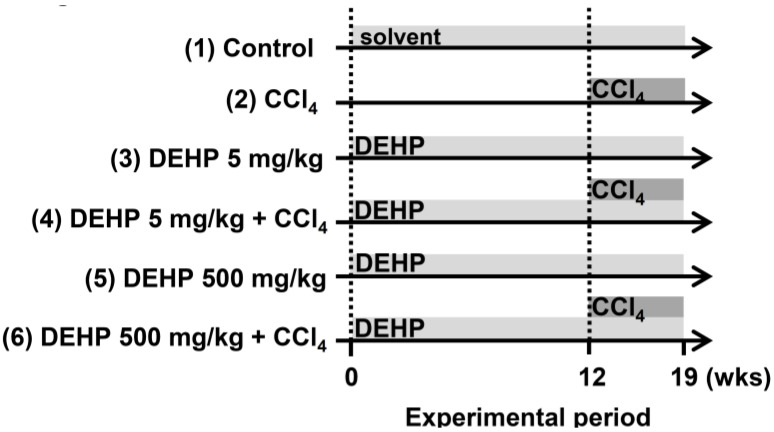
Experimental design for carbon tetrachloride (CCl_4_)-induced liver fibrosis in long-term, low-dose, di-(2-ethylhexyl) phthalate (DEHP)-exposed mice.

Male five-week-old C57BL/6 wild-type (WT) mice were purchased from the National Laboratory Animal Center, Taiwan. All mice were given a standard chow diet (no. 5001, LabDiet, St Louis, MO, USA) and were maintained in a light/dark cycle of 12 h each. As shown in Figure 1, the mice were randomly assigned to six groups: (1) the vehicle (mineral oil) control group, (2) the CCl_4_ group (2 mL/kg ([1:5 v/v in mineral oil]), intraperitoneal injection, twice weekly for 7 weeks), (3) DEHP (5 mg/kg, dissolved in mineral oil, intraperitoneal injection, six times per week for 19 weeks), (4) DEHP (5 mg/kg) plus CCl_4_ (administered as described for single-agent treatments), (5) DEHP (500 mg/kg, intraperitoneal injection, six times per week for 19 weeks), and (6) DEHP (500 mg/kg) plus CCl_4_ (administered as described for single-agent treatments). The body weight for the animals was measured once per week. At the end of the experiments, serum alanine aminotransferase (ALT) was measured with a biochemical analyzer (VetTestTM, IDEXX, Westbrook, ME, USA), and liver tissues to be used in IHC staining were fixed in 10% formalin. The experimental protocols were approved by the Institutional Animal Care and Use Committee of Taipei Medical University (LAC-2018-0008).

### 2.8. Immunohistochemical Staining and Cholesterol Quantification

Liver tissues were fixed with 4% formaldehyde and then were dehydrated by treatment with a graded ethanol series and xylene. Sirius red staining (Abcam, Cambridge, UK) and Masson’s trichrome staining (Sigma-Aldrich, St Louis, MO, USA) of paraffin-embedded liver sections (5 µm) were used to qualitatively assess collagen deposition and the extent of fibrosis, and the procedures were carried out in accordance with the manufacturer’s instructions. The intracellular concentration of cholesterol was measured using a commercial colorimetric kit (BioVision, Mountain View, CA, USA).

### 2.9. Statistical Analyses

The statistical analyses were conducted by SPSS v20.0 software (IBM, Armonk, NY, USA) and SAS 9.4 (SAS Institute Inc., Cary, NC, USA.). The Sharpio–Wilk test showed that the data fit the normal distribution. Therefore, the mean and standard deviation were reported and compared by the Student’s t-test to examine the significant differences. Differences were considered statistically significant at *p* < 0.05 (two-tailed).

## 3. Results

### 3.1. Cytotoxicity Effects of DEHP in Hepatic Stellate Cells

**Figure 2:**
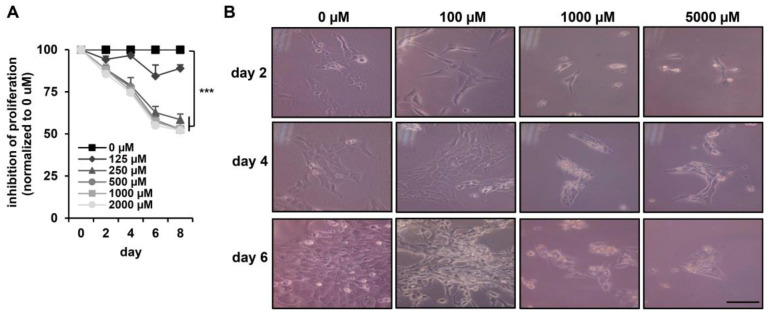
Cytotoxicity effects of DEHP in HSC-T6 cells. (**A**) HSC-T6 cells were exposed to DEHP at the indicated doses for two to eight days, and cell proliferation was assessed using an MTT assay kit. *** *p* < 0.001 vs. 0 µM. (**B**) Morphology of DEHP-treated HSC-T6 cells for two, four, and six days. Scale bar indicates 20 µm.

To determine the cytotoxic effect of DEHP in hepatic stellate cells, the viability of HSC-T6 cells was assessed with an MTT assay. As shown in Figure 2A, DEHP treatments induced a time-dependent cytotoxic effect on HSC-T6 cells. Exposure of HSC-T6 cells to DEHP (>250 µM) for two, four, six, and eight days reduced cell proliferation to 85%, 75%, 60%, and 50%, respectively, relative to the levels in untreated control cells. HSC-T6 cells continually exposed to 125 µM DEHP for eight days displayed greater than 80% viability compared to that of the untreated control (Figure 2A). HSC-T6 cell morphology before and after exposure to different concentrations of DEHP is shown in Figure 2B. At high concentrations (1000 and 5000 µM), inhibition of the cell growth effect was observed. However, treatment with 100 µM DEHP for two to six days did not result in cytotoxic effects or morphological differences compared with what was observed in control cells. These data suggest that less than 100 µM DEHP exposure did not influence acute morphology or cell growth in HSC-T6 cells. Accordingly, low doses of DEHP (50 and 100 µM) were selected for the following long-term exposure experiment in HSC-T6 cells.

### 3.2. Long-Term Exposure to Low-Dose DEHP Disturbs Cholesterol Metabolism and Synthesis in Hepatic Stellate Cells

**Figure 3:**
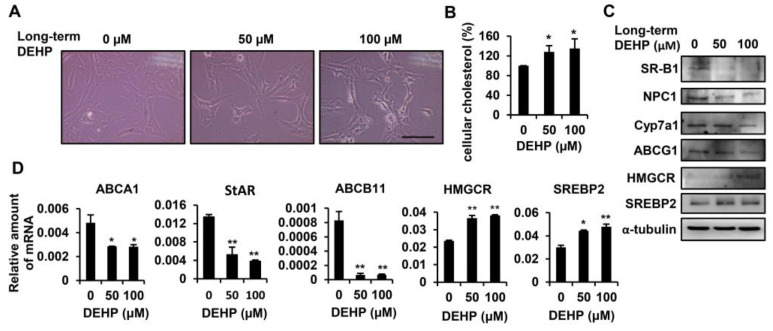
Effects of long-term exposure to low-dose DEHP on cholesterol metabolism in HSC-T6 cells. (**A**) Morphological changes induced by long-term, low-dose DEHP exposure. Scale bar indicates 20 µm. (**B**) Intracellular cholesterol content in long-term, low-dose, DEHP exposed HSC-T6 cells. (**C**) Western blot analysis of SR-B1, NPC1, Cyp7a1, ABCG1, HMGCR, SREBP2, and α-tubulin in long-term, low-dose, DEHP exposed HSC-T6 cells. (**D**) Quantitative real-time polymerase chain reaction (Q-PCR) analysis of ABCA1, StAR, ABCB11, HMGCR, and SREBP2 in long-term, low-dose, DEHP exposed HSC-T6 cells. * *p* < 0.05; ** *p* < 0.01 vs. 0 µM.

To study the effects of long-term exposure to DEHP in HSCs, HSC-T6 cells were chronically exposed to 50 and 100 µM DEHP. After 3.5 months of passage, long-term, low-dose, DEHP-exposed HSCs were obtained; they had changed morphologically into spindle-shaped cells (Figure 3A). An intracellular cholesterol quantification assay demonstrated that long-term exposure to low-dose DEHP resulted in the accumulation of cholesterol in HSC-T6 cells (Figure 3B). To clarify the molecular mechanisms responsible for cholesterol accumulation in HSC-T6 cells, we analyzed protein and mRNA expression levels for the following genes involved in different stages of the cholesterol metabolism: (1) cholesterol uptake: ATP-binding cassette A1 (ABCA1) and scavenger receptor class B type 1 (SR-B1); (2) cholesterol trafficking: Niemann–Pick type C1 (NPC1) and steroidogenic acute regulatory protein (StAR); (3) cholesterol catabolism: cholesterol 7α-hydroxylase (Cyp7a1) and ATP-binding cassette B11 (ABCB11); (4) cholesterol excretion: ATP-binding cassette G1 (ABCG1); and (5) endogenous cholesterol synthesis: 3-hydroxy-3-methyl-glutaryl-coenzyme A reductase (HMGCR) and sterol response element binding protein 2 (SREBP2). As shown in Figure 3C,D, proteins or genes involved in cholesterol uptake (SR-B1 and ABCA1), cholesterol trafficking (NPC1 and StAR), cholesterol catabolism (Cyp7a1 and ABCB11), and cholesterol efflux (ABCG1) were significantly downregulated in long-term, low-dose, DEHP-exposed HSCs. The rate-limiting enzyme for cholesterol synthesis (HMGCR) and the transcription factor that controls cholesterol homeostasis by regulating transcription of sterol-regulated genes (SREBP2) were increased in long-term, low-dose, DEHP-exposed HSCs. These data indicate that chronic low-dose DEHP exposure causes the accumulation of cholesterol in HSCs by disturbing the cholesterol metabolism and enhancing intracellular cholesterol synthesis.

### 3.3. Long-Term Exposure to Low-Dose DEHP Attenuates PDGF-BB-Induced Cell Proliferation in Hepatic Stellate Cells

**Figure 4:**
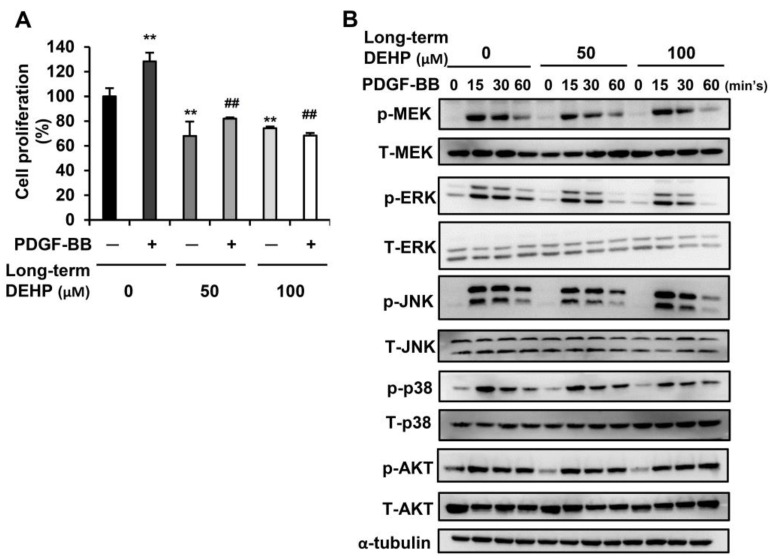
Effects of long-term, low-dose DEHP exposure on PDGF-BB induced proliferation in HSC-T6 cells. (**A**) Long-term, low-dose, DEHP-exposed HSC-T6 cells were treated with 10 ng/mL PDGF-BB for 48 h, and the cell proliferation was analyzed by MTT assay. ** *p* < 0.01 vs. black bar. ^##^ *p* < 0.01 vs. PDGF-BB-treated parental HSC-T6 cells. (**B**) Western blot analysis of total and phosphor-MEK, ERK, JNK, p38, AKT, and α-tubulin in PDGF-BB-treated long-term, low-dose, DEHP-exposed HSC-T6 cells.

Since PDGF-BB has been reported as the most potent mitogen to stimulate HSC proliferation because of its activation of the MAPK and AKT pathways [26], we next examined whether long-term exposure to low-dose DEHP affected PDGF-BB-initiated cell proliferation. As shown in Figure 4A, PDGF-BB treatment enhanced parental HSC proliferation; however, this proliferative effect was blunted in DEHP-exposed cells. In terms of the underlying molecular regulation, the phosphorylation of MEK, ERK, JNK, and p38 was decreased after 60 min of PDGF-BB treatment in DEHP-exposed cells (Figure 4B). However, there was no difference in AKT activation between PDGF-BB-treated parental and DEHP-exposed cells. These data imply that long-term, low-dose DEHP exposure in HSCs attenuates PDGF-BB-induced cell proliferation by inhibiting the MAPK pathway.

### 3.4. Long-Term Exposure to Low-Dose DEHP Triggers Apoptosis Signals in Hepatic Stellate Cells

**Figure 5:**
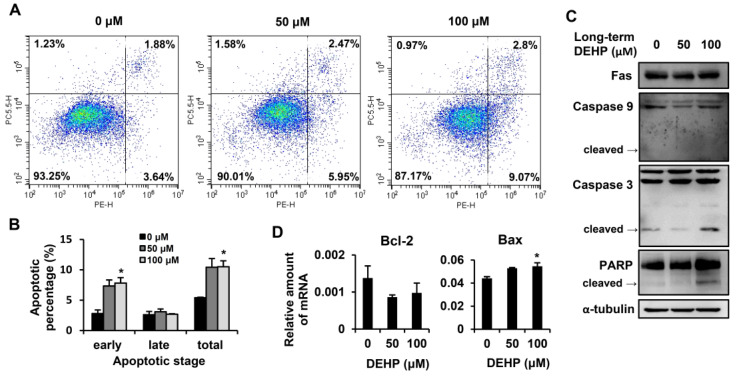
Effects of long-term, low-dose DEHP exposure-induced apoptosis in HSC-T6 cells. (**A**) Apoptosis detected by the Phycoerythrin (PE) Annexin V Apoptosis Detection Kit I and representative images are shown. Quantitative data are shown in (**B**). (**C**) Western blot analysis of Fas, Caspase 9, Caspase 3, PARP, and α-tubulin in long-term, low-dose, DEHP-exposed HSC-T6 cells. (**D**) Q-PCR analysis of Bcl-2 and Bax in long-term, low-dose, DEHP-exposed HSC-T6 cells. * *p* < 0.05 vs. 0 µM.

Next, we studied whether chronic DEHP exposure decreased cell proliferation by triggering apoptotic signaling. To evaluate the percentage of apoptotic cells, DEHP-exposed HSCs were assessed by a CytoFLEX Flow Cytometer (Figure 5A,B). The flow data revealed that, in comparison to the control, DEHP-exposed HSCs exhibited a dose-dependent increase in annexin V-FITC binding (x-axis) but only mild 7-AAD staining (y-axis), indicating that the majority of cells were undergoing early apoptosis. To further analyze the molecular changes related to this apoptotic effect, the Western blot analysis was employed, where the results showed that chronic DEHP exposure increased the levels of the cleaved form of caspase 3 and PARP (Figure 5C). However, the expression of FAs and the cleaved form of caspase 9 did not increase in DEHP-exposed HSCs. The mRNA level of the pro-apoptotic Bax gene was upregulated in DEHP-exposed HSCs (Figure 5D), but DEHP exposure did not alter the expression of the anti-apoptotic gene Bcl-2 in HSCs. These data indicate that the inhibition of cell proliferation in long-term, low-dose, DEHP-exposed HSCs arises primarily from blocking the PDGF-BB/MAPK pathway and from inducing the caspase 3/PARP-dependent apoptotic signal.

### 3.5. Long-Term Exposure to Low-Dose DEHP Impairs Mitochondrial Respiration Function in Hepatic Stellate Cells

**Figure 6:**
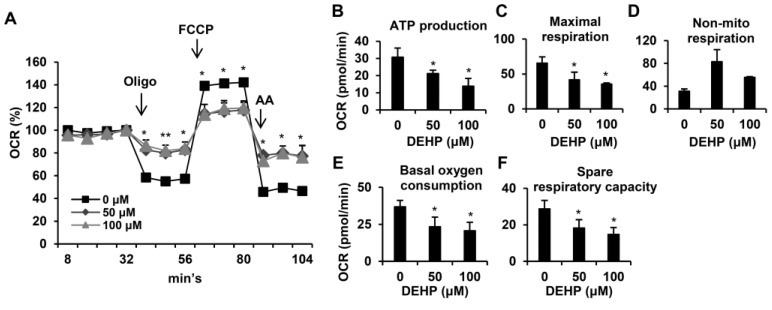
Effects of long-term, low-dose DEHP exposure-induced mitochondrial dysfunction in HSC-T6 cells. (**A**) Mitochondrial respiration functions were analyzed by the Seahorse XFe Extracellular Flux Analyzer. Oligomycin (Oligo, 1 μM), FCCP (2 μM) and rotenene/antimycin A (AA, 0.5 μM) were injected into the well at the fourth, eighth, and 11th time point. Quantitative data are shown in (**B**–**F**). * *p* < 0.05; ** vs. 0 µM.

Since mitochondria play a key role in cellular metabolism [27], which controls cell growth and apoptosis [28], we next examined whether chronic DEHP exposure affected mitochondrial respiration in HSCs (Figure 6A). Normalized at the fourth point, the oxygen consumption rates (OCR) were detected by XF24 bioenergetic assays. Oligomycin was used to inhibit ATP synthase, FCCP was used to assess the maximal oxygen consumption, and a mixture of rotenone and antimycin A was used to block the electron transport pathway. Experimental data showed that chronic DEHP-exposed HSCs had an OCR that was mildly reduced after the addition of oligomycin, indicating that DEHP-exposed HSCs had a reduced ability to produce ATP (Figure 6B). Compared to the control, cells exposed to DEHP exhibited barely elevated OCR following FCCP injection, indicating that the mitochondrial maximal working capacity was impaired (Figure 6C). By adding rotenone and antimycin A, the OCR of DEHP-exposed HSCs could not return to the baseline, which revealed that nonmitochondrial respiration occurred during the reaction (Figure 6D). In addition, the level of spare respiratory capacity, which is representative of the ability to manage acute stress demands, as well as basal oxygen consumption decreased in chronic DEHP-exposed HSCs (Figure 6E,F). These data suggest that long-term exposure to low-dose DEHP induces mitochondrial dysfunction and may influence apoptosis in HSCs.

### 3.6. Chronic Long-Term Exposure to Low-Dose DEHP Accelerates CCl_4_-induced Liver Damage and Fibrosis in Mice

**Figure 7:**
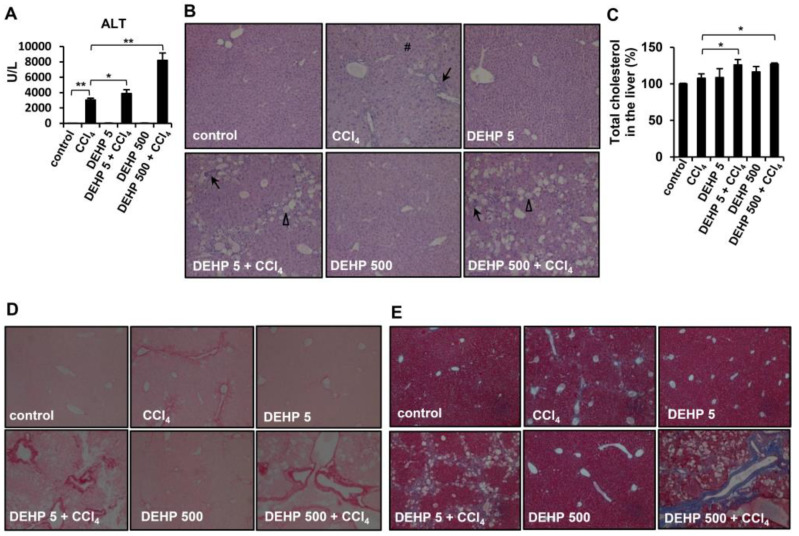
Effects of long-term, low-dose DEHP exposure in CCl_4_-treated mice. (**A**) Serum alanine aminotransferase (ALT). (**B**) Hematoxylin and eosin (H&E) staining of liver tissues Δ, fatty change #, perinuclear vacuoles ↑, and inflammatory infiltration. (**C**) Hepatic cholesterol content. (**D**) Sirius red, and (**E**) Masson’s Trichrome staining of liver tissues. * *p* < 0.05; ** *p* < 0.01.

To further investigate whether chronic DEHP exposure affects liver fibrosis progression in vivo, CCl_4_ injections were used to induce liver fibrosis in mice. As shown in Figure 7A, serum ALT levels were increased in CCl_4_-treated mice compared to those of vehicle control mice. Notably, chronic DEHP exposure significantly increased CCl_4_-induced serum ALT levels. However, DEHP exposure alone for 19 weeks did not alter the serum ALT levels. Analysis of the liver pathology revealed inflammatory infiltration and perinuclear vacuoles in CCl_4_-treated mice (Figure 7B). Although DEHP exposure alone did not cause any pathological damage in the liver, more severe fatty changes, irregular infiltration border perinuclear vacuoles, and regional inflammation were observed in the liver tissues of DEHP plus CCl_4_-treated mice than in the control mice (Figure 7B). In addition, long-term exposure to low-dose, DEHP-enhanced hepatic cholesterol accumulation was observed in CCl_4_-treated mice (Figure 7C). Collagen deposition, an indicator of liver fibrosis, was assessed by Sirius red and Masson’s trichrome staining. As shown in Figure 7D,E, collagen deposition was observed in CCl_4_-treated mice; notably, this phenomenon was more severe in the chronic DEHP exposure plus CCl_4_-treated mice. These data indicate that long-term exposure to low-dose DEHP accelerates CCl_4_-induced liver damage and fibrosis in mice.

## 4. Discussion

**Figure 8:**
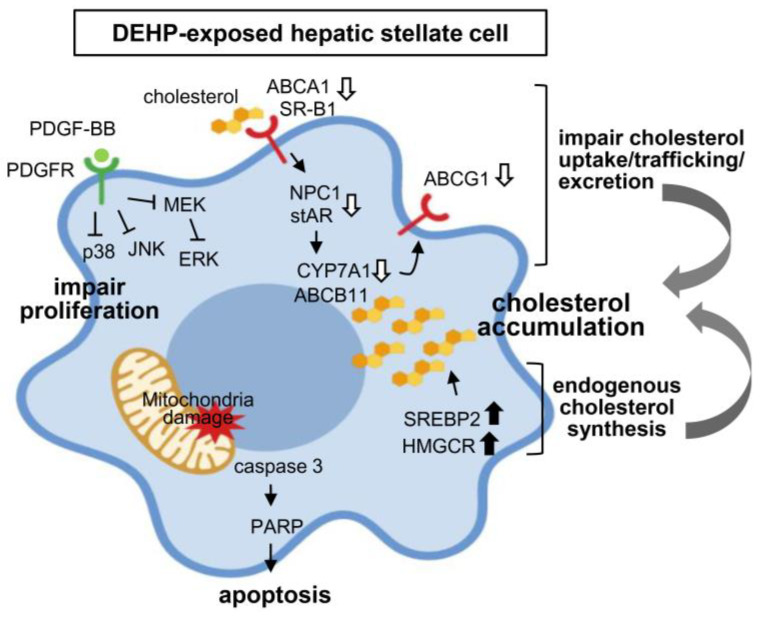
Proposed mechanisms of long-term, low-dose DEHP exposure on hepatic stellate cell (HSC) activation and liver fibrosis.

This study has provided experimental evidence showing the harmful effects of long-term exposure to low-dose DEHP in deregulating the cholesterol metabolism and mitochondrial dysfunction-induced proliferation/apoptosis imbalance in HSCs, thereby contributing to the progression of liver fibrosis (Figure 8).

The liver is composed of several kinds of cells, and the sensitivity of each cell type to DEHP is quite different. In rat hepatocytes, 200 μM of DEHP exposure inhibited 50% of cell growth [19]. The calculated IC_50_ of DEHP was 536 μM in rat FL83B hepatocytes [29]. Our study showed that a lower dose of DEHP (<250 μM) barely inhibited HSC growth; however, the proliferation rate gradually decreased over a course of days with a higher dose of DEHP (>250 μM). These data suggest that different types of liver cells exhibit different DEHP sensitivities. These data indicate that different liver cells express individual sensitivity to DEHP exposure. Notably, our study demonstrated that long-term exposure to low-dose DEHP changed HSC morphology into spindle-shaped cells, which indicated a transformation into myofibroblasts [30]. Regarding the in vivo DEHP exposure dosage, due to the different amounts of DEHP released from food or water, we were able to estimate the exposed ranges of DEHP. The new U.S. Environmental Protection Agency (EPA) Risk Assessment Guidelines showed that the daily level of DEHP exposure in the general population is in the range of 3–30 μg/kg from food (4.9–18 μg/kg), water, and the environment. In the clinical setting, patients may suffer a dosage of 28.4–94.6 μg/kg from respiratory tubes, 30 μg/kg from nutrient bags, and 457 μg/kg from intravenous tubes in haemodialysis patients [12,31]. Due to the regular exposure of DEHP in haemodialysis patients, we consider 457 μg/kg as a low-dose exposure to DEHP in our study design and convert the value to approximately 5 mg/kg in mice by applying the equation from Reagan–Shaw et al. [32]. In 2011, explosive news on the incidence of plasticizer-contaminated foodstuffs was reported in Taiwan [33,34]. Overseers of Taiwanese food containers and appliances stipulate that the concentration of DEHP which migrated from plastic items cannot exceed 1.5 ppm. However, the Taiwan Food and Drug Administration discovered that up to 7% of plastic containers exceeded the standard, and the released level of DEHP was mostly within 1–100 ppm [34]. Therefore, we chose the middle level of 40 ppm in plastic items, [31] which, converted to a dose of 500 mg/kg in mice, represents high DEHP dose exposure. As compared with Zhao et al., proposed that the serum level of 500 mg/kg DEHP-exposed mice was 0.65 μg/mL [3], which is relevant to the serum level of 0.5–0.8 μg/mL in long-term haemodialysis patients [35]. These differences may refer to the method of detecting DEHP. Zhao et al. compared the serum levels of DEHP, while in present study, we compared the intake levels. Because of the complicated metabolic routes and several metabolites of DEHP, it is hard to draft the actual reaction to DEHP exposure. Importantly, our data showed that long-term exposure to both lower and higher doses of DEHP for 19 weeks accelerated CCl_4_-induced liver damage and fibrosis compared to that of CCl_4_ treatment alone in mice. This phenomenon was also observed in Sprague-Dawley (SD) rats following nine weeks of DEHP exposure [36].

The liver is the main organ involved in cholesterol homeostasis [37]. In humans, higher dietary consumption of cholesterol is associated with a higher risk of cirrhosis or liver cancer [38]. Several in vivo studies have found that a high-cholesterol diet exacerbates CCl_4_, and that bile duct ligation induced liver fibrosis in rodents [39,40]. Dysregulated cholesterol metabolism also increases the sensitivity of HSCs to TGF-β [25,40]. Our study showed that severe steatosis and increased hepatic cholesterol levels were observed in chronic DEHP-exposed HSCs and mice (Figure 3 and Figure 7). Zhao et al. found hypercholesterolemia and fatty livers in DEHP-exposed, apolipoprotein-E-deficient mice [3], implying that DEHP exposure may disturb the cholesterol metabolism. Regarding the mechanisms of long-term, low-dose, DEHP exposure-induced cholesterol accumulation, we showed that chronic exposure to low-dose DEHP decreased the levels of the cholesterol excretion receptor [41,42] ABCG1. NPC1 and StAR, which are endosomal cholesterol trafficking proteins and mitochondrial cholesterol transport proteins, carry cholesterol to the endoplasmic reticulum and other membranes [41,43]; these proteins were downregulated in DEHP-exposed HSCs. Moreover, excessive free cholesterol can be diverted in a rate-limiting fashion to bile acid by Cyp7a1 [41,42,44], or it can be excreted by ABCB11 [41,43,45,46,47]; these factors were also decreased in DEHP-exposed HSCs. On the other hand, DEHP exposure-enhancing endogenous cholesterol synthesis is one of the major reasons for cholesterol accumulation because it increases the expression of HMGCR and SREBP2. HSC is the major cell which promotes the pathogenesis of liver fibrosis via producing extracellular matrix (ECM) proteins in the damaged liver [24]. We found that the expression of matrix metalloproteinase 2 (MMP2), tissue inhibitors of metalloproteinases (TIMP), and desmin did not change in DEHP-treated hepatic stellate cells (Figure S1). These in vitro data are similar to that of long-term DEHP-exposed mice. In Figure 7D,E, the mice exposed to 5 and 500 mg/kg of DEHP alone did not induce collagen deposition in the liver tissues. However, under CCl_4_ treatment, collagen deposition was more severe in the chronic DEHP-exposed mice. These data suggest that although long-term exposure to low-dose DEHP alone did not induce ECM-related gene expression, under some liver damage stimuli, DEHP exposure can accelerate liver damage and fibrosis.

Taken together, long-term exposure to low-dose DEHP was not only found to impair cholesterol uptake/trafficking/excretion, but also increased endogenous cholesterol synthesis, which may contribute to HSC activation and promote liver fibrosis.

## 5. Conclusions

In conclusion, this study provides experimental evidence showing the harmful effects of long-term exposure to low-dose DEHP in deregulating cholesterol metabolism and mitochondrial dysfunction-induced proliferation/apoptosis imbalance in HSCs, thereby contributing to the progression of liver fibrosis.

## Figures and Tables

**Table 1:** Primers used for real-time PCR.

Gene	Forward Sequences (5’–3’)	Reverse Sequences (5’–3’)
Bcl-2	AGGAAGTGAACATTTCGGTGAC	GCTCAGTTCCAGGACCAGGC
Bax	GATCCAGGATCGAGCAGA	AAGTAGAAGAGGGCAACCAC
ABCA1	CCCCTGCTTCCGTTATCCA	GGACCTTGTGCATGTCCTTAATG
ABCB11	CAGAACATGACAAACGGAACAAG	CCTGCGTATGCCAGAAAATT
HMGCR	TGTGGTTTGTGAAGCCGTCAT	TCAACCATAGCTTCCGTAGTTGTC
SREBP2	CAGACAGCCGCCCTTCAAGT	GCTGTTCATTGACCTTCTCCCG
GAPDH	TCACCACCATGGAGAAGGC	GCTAAGCAGTTGGTGGTGCA

## Supplementary Materials

The following are available online at https://www.mdpi.com/1660-4601/17/11/3802/s1, Figure S1. Q-PCR analysis of MMP2, TIMP, and desmin in long-term, low-dose DEHP exposed HSC-T6 cells.
