# Supplementary material for: Long-Term Exposure to Low-Dose Di-(2-ethylhexyl) Phthalate Impairs Cholesterol Metabolism in Hepatic Stellate Cells and Exacerbates Liver Librosis
Source: Int J Environ Res Public Health. 2020 May 27;17(11):3802. doi: 10.3390/ijerph17113802 (PMC7312183; doi:10.3390/ijerph17113802)
Supplement: Supplementary file 1 [file ijerph-17-03802-s001.pdf]

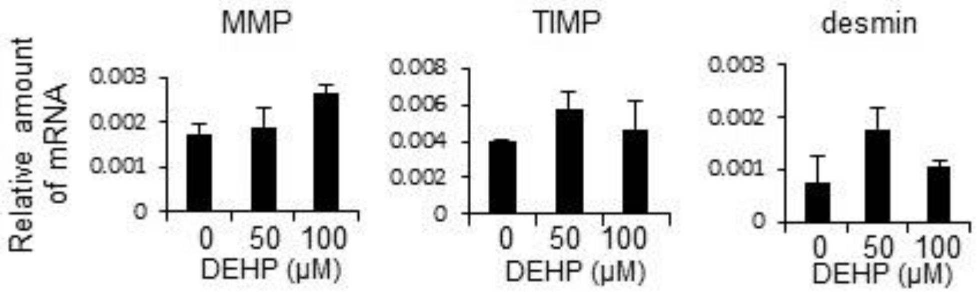

Supplementary Figure 1. Q-PCR analysis of MMP2, TIMP, and desmin in long-term, low-dose DEHP exposed HSC-T6 cells.
